# Kinematic Parameters of Normal Hand-to-Mouth Movement in Pediatric Populations: Adaptation of the “Rab Hand-to-Mouth Protocol”

**DOI:** 10.3390/s26092625

**Published:** 2026-04-23

**Authors:** Álvaro Pérez-Somarriba Moreno, Rosa María Ortiz-Gutiérrez, Patricia Martín-Casas, Iñigo Monzón Tobalina, Paula Arias Martínez, Ignacio Martínez Caballero, Angélica Guerrero-Blázquez, María José Díaz-Arribas

**Affiliations:** 1 Doctoral Program in Healthcare, Faculty of Nursing, Physiotherapy and Podiatry, Universidad Complutense de Madrid, Plaza de Ramón y Cajal, s/n, 28040 Madrid, Spain; aperezso@ucm.es (Á.P.-S.M.); angeli06@ucm.es (A.G.-B.); 2 Department of Physiotherapy, Faculty of Nursing, Physiotherapy and Podiatry, Universidad Complutense de Madrid, Plaza de Ramón y Cajal, s/n, 28040 Madrid, Spain; pmcasas@ucm.es (P.M.-C.); mjdiazar@ucm.es (M.J.D.-A.); 3 Comprehensive Integral Rehabilitation, Physiotherapy and Neurorehabilitation Research Group, Universidad Complutense de Madrid, Plaza de Ramón y Cajal, s/n, 28040 Madrid, Spain; 4 InPhysio Research Group, Health Research Institute of the Hospital Clínico San Carlos (IdISSC), C/Prof Martín Lagos, s/n, 28040 Madrid, Spain; 5 Clinical Motion Analysis Laboratory, Niño Jesús Children’s Hospital, 28009 Madrid, Spain; imonzontobalina@gmail.com; 6 Research Foundation of the Niño Jesús Children’s Hospital, 28009 Madrid, Spain; paularimart@gmail.com; 7 Department of Traumatology, Niño Jesús Children’s Hospital, 28009 Madrid, Spain; manatina.martinez@gmail.com

**Keywords:** motor skills, upper extremity, kinematics, pediatrics

## Abstract

Optoelectronic motion capture systems provide objective and high-resolution measurements of upper limb kinematics. The hand-to-mouth movement is closely related to motor development in children. The “Rab Hand-to-Mouth protocol” (BTS Bioengineering) is widely used; however, its seated configuration constrains elbow posture and may limit the ecological validity of the movement. In this study, we propose a methodological adaptation of the protocol in a standing position to allow a more physiological elbow configuration and to increase the dynamic range of elbow and shoulder motion. The objective was to characterize kinematic patterns of the hand-to-mouth movement in typically developing children aged 4 to 9 years using this adapted setup. This study was designed as a descriptive analysis and does not aim to provide formal validation of the standing protocol against the original seated configuration. An observational study that included 40 children was conducted. Motion data were acquired using eight optoelectronic cameras (sampling frequency: 250 Hz) and 17 reflective markers placed on the trunk and upper limbs. Kinematic patterns and spatiotemporal parameters were computed using dedicated motion analysis software. No significant differences were observed between dominant and non-dominant limbs in spatiotemporal parameters, whereas kinematic differences were minimal and limited to trunk rotation, as identified by Statistical Parametric Mapping (SPM). Some isolated statistically significant associations with age were identified in specific spatiotemporal variables; however, these variables showed low coefficients of determination (R^2^), indicating limited explanatory power of age. Overall, kinematic parameters did not exhibit consistent age-related patterns. These findings provide preliminary descriptive data for hand-to-mouth kinematics in a standing condition, which may contribute to the future development of assessment protocols. However, the limited sample size and the absence of pathological populations restrict the direct generalization of these findings. Future studies should evaluate the applicability of this approach in clinical cohorts and explore its integration into sensor-based and data-driven models for movement analysis.

## 1. Introduction

Quantitative assessment of upper limb kinematics using optoelectronic motion capture systems has become a well-established approach for the objective evaluation of motor function [1]. Despite this technological advancement, the clinical interpretation of kinematic data remains dependent on standardized protocols and task configurations [2]. The Rab Hand-to-Mouth protocol is widely used for upper limb assessment; however, its seated configuration can influence elbow position and limit the acquisition of proximal kinematic compensations, especially in the pediatric population [3]. This methodological limitation may reduce the functional representativeness of the task and affect the interpretation of normative kinematic values [4].

The hand-to-mouth gesture is one of the earliest goal-directed upper limb movements acquired during early childhood development and represents an important indicator of the early integration of motor and sensory systems [5]. This gesture can be defined as an ecological and standardized functional task consisting of bringing the hand or an object from an initial position to the mouth and subsequently returning to the starting position, with the mouth acting as a spatial target that guides motor planning and execution rather than an anatomical structure actively involved in the movement itself [6].

The acquisition of the hand-to-mouth gesture is modulated by neurological maturation, the environmental context, and sensorimotor experiences, and it is closely associated with functional independence in feeding and self-care activities [7]. Consequently, impairments in this movement may occur in children with neurodevelopmental disorders, potentially affecting activities of daily living (ADLs), participation, and quality of life. Therefore, the analysis of this gesture may provide relevant insights into motor development [3].

Three-dimensional motion analysis (3DMA) is considered a reference method to quantitatively assess motor function [8]. The analysis of upper limb movement allows for the acquisition of precise, objective, and reproducible measurements of functional performance during ADLs, such as the hand-to-mouth gesture, treatment planning, and follow-up in pediatric populations with movement disorders [9,10].

In contrast to pediatric gait analysis, which is widely used to quantify locomotor impairments and guide therapeutic decision-making, the application of 3DMA to the upper limb remains more challenging. This increased complexity is mainly related to the anatomical characteristics of the upper limb and the high degree of inter-joint coordination required to perform functional tasks [7].

For the 3DMA of the hand-to-mouth gesture, the “Rab Hand-to-Mouth” protocol, developed by BTS Bioengineering (Italy), is the most widely used kinematic protocol among the motion analysis suite. It is based on the model developed by Dr. George Rab, Kyria Petuskey, and Anita Bagley at Shriners Hospital for Children, Northern California. This protocol proposes a standardized kinematic model, including a predefined initial position of the gesture and the placement of passive reflective markers on specific anatomical landmarks of the head, trunk, and upper limbs. Using three-dimensional optoelectronic motion capture systems, the protocol enables segmental recording of kinematic and spatiotemporal variables during task execution [11,12,13,14].

In general, the “Rab Hand-to-Mouth protocol” defines the initial position as sitting, with the hand resting on the thigh or on a table and the trunk aligned. In this position, the elbow starts the movement in slight flexion, the forearm is in a neutral position or slight pronation, and the wrist is in a neutral position or slight extension, while the hand and fingers remain passively supported [4,14]. From a biomechanical and functional perspective, this initial posture is commonly considered physiological, as it closely resembles the resting position of the upper limb during sitting. Moreover, no joint starts from an extreme range of motion, which minimizes passive joint and muscle tension [15].

However, from an anatomical and biomechanical perspective, analyzing the hand-to-mouth gesture from a standing position entails greater functional demands [7,16]. Previous studies on the 3DMA of functional reaching tasks in pediatric populations have shown that the initial position and the magnitude of elbow flexion have direct implications for kinematic parameters such as movement range and movement duration [17]. Similarly, performing the analysis while standing allows the evaluation of the gesture while incorporating trunk involvement in postural control [18,19]. Unlike the seated position, raising the arm and directing it toward the mouth from a standing position require continuous dynamic adjustments to maintain postural stability [20].

In this context, adapting the protocol to a standing configuration may allow the exploration of a wider range of movement strategies and a more physiological elbow position during task execution. Rather than replacing the original protocol, this approach aims to complement existing methodologies by providing additional kinematic information under functionally relevant conditions [21]. Furthermore, the generation of normative kinematic datasets under these conditions may be of interest for future applications involving wearable sensors (e.g., inertial measurement units) and data-driven approaches, where ecologically valid movement patterns are required.

The objective of this study was to generate descriptive reference data of the hand-to-mouth movement in typically developing children aged 4 to 9 years using an adapted standing version of the “Rab Hand-to-Mouth protocol”. In addition, this study aimed to examine potential differences between the dominant and non-dominant upper limbs to determine whether lateralized motor patterns are already present during this stage of development. These results are intended to provide preliminary reference data that may support future comparative studies, including those involving pediatric populations with neurodevelopmental disorders [7,14,22].

## 2. Materials and Methods

### 2.1. Study Design and Ethical Statement

The study protocol of this descriptive cross-sectional study was approved by the Ethics Committee of The Niño Jesus Hospital, Madrid, Spain (protocol code R-0091/24). The legal guardians of all participants provided written informed consent prior to their inclusion, in accordance with the Declaration of Helsinki (revised 2024). Patient privacy and data confidentiality were ensured throughout the study.

### 2.2. Sample

Participants were recruited using non-probability convenience sampling. Eligible participants were children of both sexes between the ages of 4 and 9 with no known or diagnosed neurological, metabolic, or musculoskeletal disorders. They also had to have no orthopedic history that could affect upper limb movement or their ability to cooperate with the study requirements.

### 2.3. Data Collection and Outcome Measures

Basic demographic variables, including age and sex, were recorded. Hand dominance was determined using the Edinburgh Handedness Inventory (EHI) [23].

**Figure 1:**
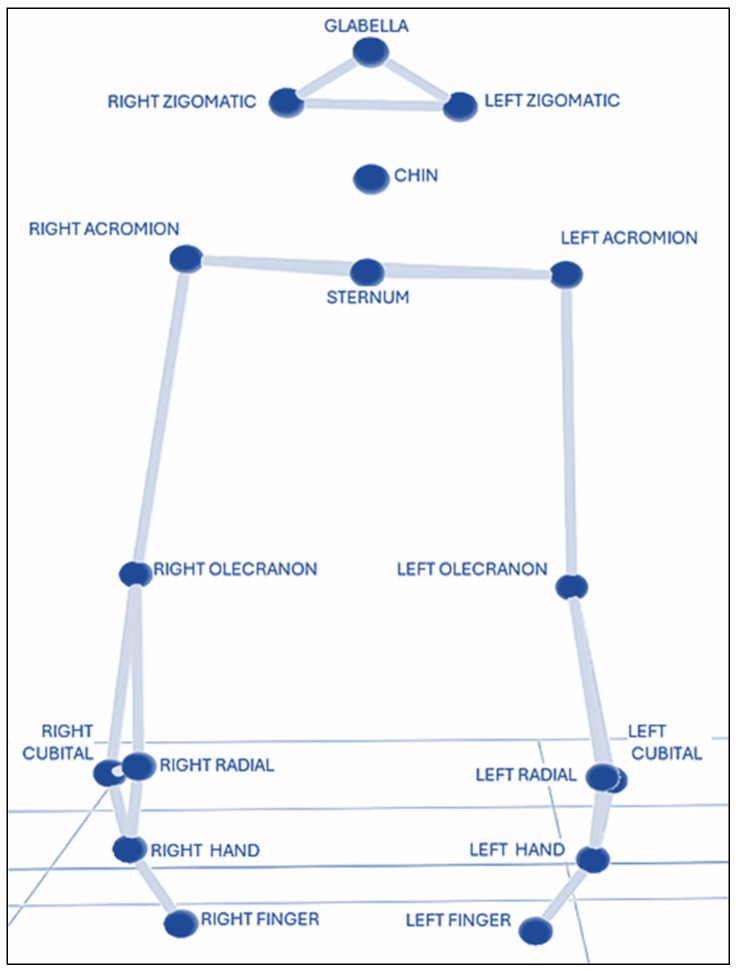
Markers of the “Rab Hand-to-Mouth” kinematic model in the static position.

All participants underwent 3DMA using the “Rab Hand-to-Mouth kinematic protocol” from BTS Bioengineering (Italy) [11,12], which was adapted from the original sitting posture to a standing position. Participants stood upright in a natural posture, with their feet together or slightly apart, arms resting alongside the body, shoulders in a neutral position, elbows in slight flexion, forearms in a neutral position between pronation and supination, and the hands positioned adjacent to and lightly resting against the lateral aspect of the thighs. Seventeen reflective markers, all 1.5 cm in diameter, were placed on anatomical landmarks as follows: the head (glabella, right and left zygomatic arches, and chin); the trunk (suprasternal notch and right and left acromion); bilaterally on upper limbs (olecranon and radial and ulnar styloid processes); and bilaterally on hands (the dorsal aspect of the third metacarpal head and the nail of the index finger) (Figure 1). Marker placement was only performed by a single trained evaluator following the standard BTS protocol.

After a static calibration trial, two dynamic acquisitions of the hand-to-mouth gesture were performed. In each acquisition, six repetitions were requested, starting with the dominant hand at a self-selected speed and alternating between the dominant and non-dominant sides [22,24]. Participants were instructed as follows: “Touch your mouth with the tip of your index finger, as if giving a kiss, and return to the starting position next to your thigh.” Two practice trials were allowed to familiarize participants with the procedure, and they were asked to keep their gaze fixed on a point located in front of them [13]. For each trial, the participant was instructed to repeat the hand-to-mouth gesture three consecutive times per side.

The initial position was defined as the index finger resting near the lateral aspect of the thigh, with the movement directed toward the mouth as the spatial target. Movement onset and termination were automatically detected by the software based on kinematic changes and manually verified by the evaluator.

Movement phases were defined based on the kinematic criteria derived from the velocity of the index finger marker. Three phases were identified: Going, Adjusting, and Returning. The Going phase was defined as the period beginning when the velocity of the index finger marker exceeded 50 mm/s and ending when the velocity dropped below this threshold. The Adjusting phase was defined as the period beginning when the velocity of the index finger marker fell below 50 mm/s following the Going phase, corresponding to the fine motor adjustment of the finger near the target. The Returning phase was defined as the period beginning after the Adjusting phase, when the finger moved away from the target, and ending when the velocity of the index finger marker decreased below 50 mm/s.

Motion data were captured using eight high-speed infrared digital optoelectronic cameras (BTS Bioengineering, Milan, Italy) operating at a sampling frequency of 250 Hz and filtered at 4 Hz with a low-pass Butterworth filter [14]. Data processing was performed using Smart Clinic software (Origen Biomedica, version 1.10/14/110/2022), which included manual identification of each marker and automatic detection of movement events; all automatically detected movement events were visually inspected and verified by the evaluator to ensure accuracy. Trials were selected based on the technical adequacy of the recording (e.g., complete marker visibility, correct movement capture, etc.). Trials and consecutive repetitions within patients for the hand-to-mouth gesture were averaged to obtain a single representative waveform per patient and side.

Spatiotemporal event parameters were identified and analyzed for each side, as defined in Table 1. Additionally, the maximum, minimum, and range-of-motion (ROM) values were calculated for each joint.

### 2.4. Statistical Analysis

Kinematic outcome measures included the maximum, minimum, and range-of-motion (ROM) values for each joint.

Statistical Parametric Mapping (SPM) was employed to assess differences across the entire normalized movement cycle, enabling the analysis of continuous kinematic time-series data and increasing sensitivity to detect subtle, time-dependent variations in complex motor patterns [25,26].

In addition, ordinary least squares (OLS) linear regression models were applied to investigate the associations between age and spatiotemporal as well as kinematic parameters. For each model, the annual rate of change (slope), its statistical significance, 95% confidence intervals, and the coefficient of determination (R^2^) were estimated.

Statistical comparative analyses were conducted using Python Software Foundation’s Python Language Reference version 3.11 (https://www.python.org) [27] and its scientific computing ecosystem, including the NumPy [28], Pandas, SciPy [29], spm1d, Matplotlib, and Seaborn libraries (version 0.11.x from 2021) [30]. Data normality was assessed using the Shapiro–Wilk test. Side-to-side comparisons were performed using a paired Student’s t-test when normality assumptions were satisfied or the Wilcoxon signed-rank test when normality was violated. Statistical significance was set to *p* < 0.05 [31].

All kinematic and spatiotemporal variables were averaged at the participant level prior to statistical analysis. Specifically, repetitions from both dynamic acquisitions were pooled and averaged to obtain a single representative value per participant and limb. Each participant was treated as the statistical unit of analysis. No correction for multiple comparisons was applied due to the descriptive nature of the study.

## 3. Results

### 3.1. Descriptive Analysis

A total of 40 typically developing (TD) children participated in this study, including 21 boys (52.5%) and 19 girls (47.5%). The mean age of the participants was 7.12 ± 1.59 years. With respect to handedness, three participants were left-handed (7.5%), while 37 were right-handed (92.5%).

### 3.2. Normality Plots of the Hand-to-Mouth Movement and SPM

**Figure 2:**
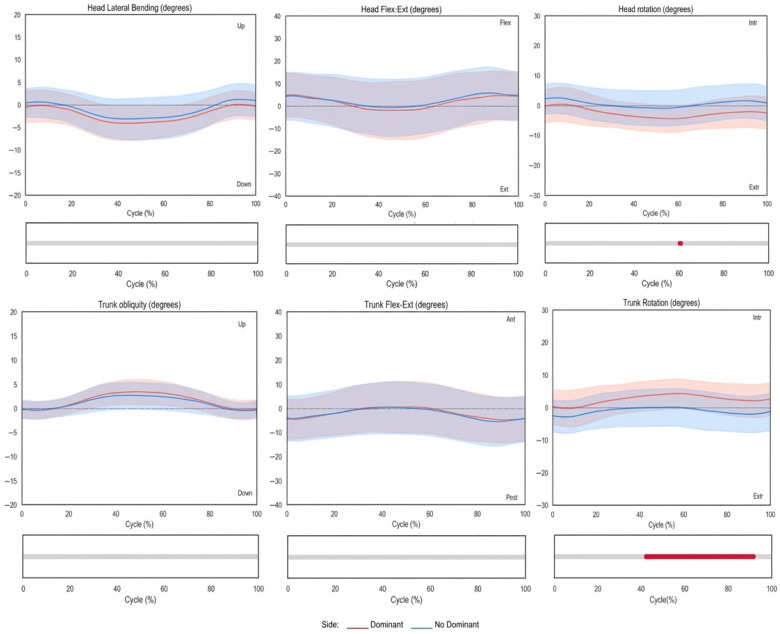
Trunk and head kinematics during the hand-to-mouth task. Shaded bands indicate variability (wider = greater inter-subject variability). Lower grey bars show symmetry between sides; the red segment marks statistically significant differences.

**Figure 3:**
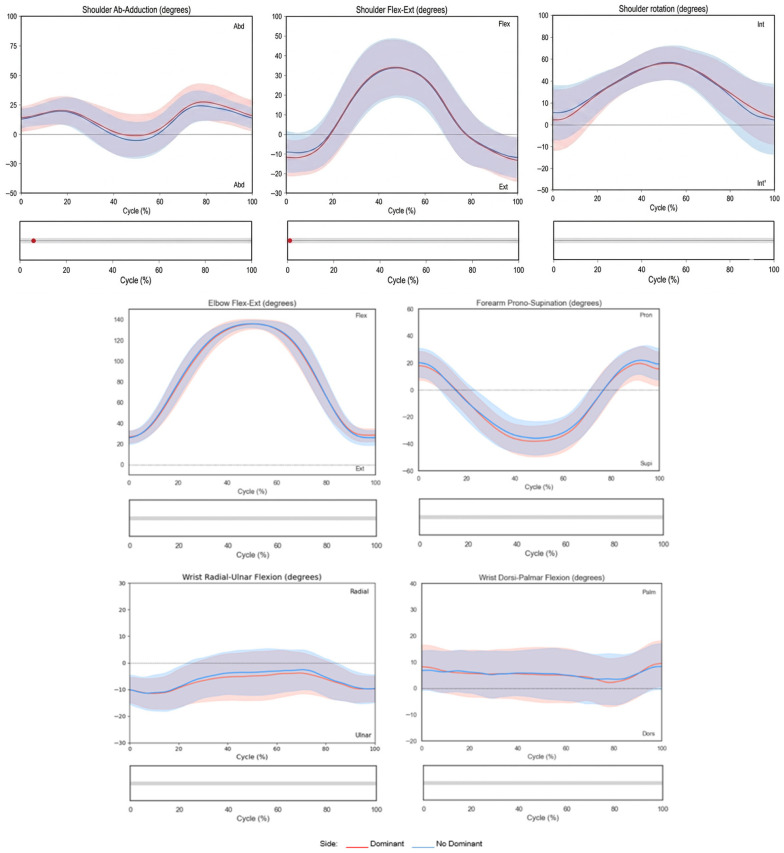
Shoulder, elbow, forearm and wrist kinematics during the hand-to-mouth task. Shaded bands indicate variability (wider = greater inter-subject variability). Lower grey bars show symmetry between sides.

Figure 2 and Figure 3 illustrate the joint angles plotted over the normalized movement cycle (0–100%). The *x*-axis represents the movement cycle (%), and the *y*-axis represents joint angles (°). Shaded bands indicate standard deviations (SDs). The dominant side is shown in red, and the non-dominant side is shown in blue. Statistically significant differences (*p* < 0.05) detected by SPM are highlighted in red below each plot.

In the trunk and head kinematics during the hand-to-mouth task shown in Figure 2, the head exhibits a slight ipsilateral tilt and rotation toward the moving side, with extension during the forward movement and gradual return during the return phase. The trunk showed slight elevation, antepulsion, and internal rotation of the ipsilateral hemithorax during the forward phase, peaking during the adjustment phase and returning to the initial position afterward. Significant trunk rotation differences between sides were observed during the adjustment and return phases, with the non-dominant side showing slight external rotation relative to the spinal axis and the dominant side showing internal rotation (Figure 2).

In relation to upper limb kinematic analysis, the shoulder initiates the movement in slight abduction, extension, and internal rotation, increasing flexion and internal rotation during the forward phase with slight final adduction. The elbow moves from 25° to 136° of flexion. The forearm progresses from 24° pronation to 40° supination. The wrist maintained a reduced range of motion with slight palmar flexion, and values ranging from approximately 2° to 13° and an ulnar deviation of around 10° were observed during the entire task (Figure 3).

**Figure 4:**
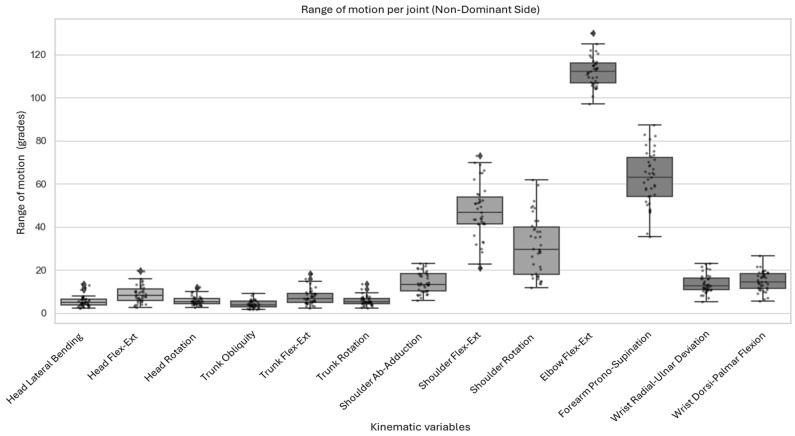
Range of motion of each joint for each movement on the non-dominant side. Dots represent each subject’s measured value; Diamonds indicate the mean value for each kinematic variable.

The largest ranges of motion were observed in elbow flexion–extension, forearm pronation–supination, and shoulder flexion and rotation. In contrast, head, trunk, and wrist movements exhibited limited mobility, with ROM values below 20°. Figure 4 summarizes the ROM values for the non-dominant side.

### 3.3. Comparison of Spatiotemporal and Kinematic Parameters Between the Dominant and Non-Dominant Sides

Comparisons between the dominant and non-dominant sides revealed no statistically significant differences in any of the spatiotemporal parameters.

Among the kinematic variables, only a limited number of statistically significant differences were observed between sides, indicating that the hand-to-mouth gesture is performed in a largely symmetrical manner (Table 2). Results should therefore be interpreted descriptively rather than inferentially.

For head movements, differences were detected in the minimum values of flexion–extension and rotation, with slightly greater displacement observed on the dominant side. No significant differences were found in the corresponding maximum values or in the overall ROM, suggesting that these variations represent minor postural adjustments rather than meaningful kinematic asymmetrical.

For the trunk, between-side differences were only observed for the ROM of obliquity, as well as in the maximum and minimum values of axial rotation, which were greater on the dominant side. Nevertheless, these differences were small in magnitude and were not accompanied by significant changes in the ROM in the remaining planes of motion.

At the wrist, a between-side difference was only observed for the ROM of radial-ulnar deviation, with a slightly greater ROM on the non-dominant side. No side-to-side differences were observed for flexion–extension.

Overall, these findings indicate that, despite the presence of small and localized differences at the head, trunk, and wrist levels, the hand-to-mouth movement is predominantly symmetrical.

### 3.4. Spatiotemporal and Kinematic Differences According to Age

Linear regression analysis indicated that, within the 4–9-year age range, age was not a strong explanatory variable for the variability observed in most spatiotemporal and kinematic parameters of the hand-to-mouth gesture. For most variables, *p*-values exceeded the significance threshold (*p* > 0.05), and coefficients of determination (R^2^) remained consistently low (0.00–0.15), indicating limited predictive power of age with respect to movement performance (Table 3).

For spatiotemporal parameters, no significant associations with age were observed for cycle time, the number of movement units, the curvature index, or asymmetry, suggesting relative stability in the temporal organization and geometric structure of the gesture across this developmental interval. The Going phase showed a statistically significant age-related trend only on the dominant side (*p* = 0.022, R^2^ = 0.131), while force and resistance exhibited isolated significant associations on the non-dominant side (*p* = 0.008 and *p* = 0.038, respectively), albeit with limited explanatory strength. The average jerk and mean movement velocity demonstrated positive associations with age on both sides, with values being more pronounced on the non-dominant side, where R^2^ values reached up to 0.285, indicating a gradual increase in movement smoothness and execution speed with age. These findings indicate that, despite reaching statistical significance in some cases, age explains only a small proportion of the variability in the analyzed parameters, and therefore these associations should be interpreted with caution.

With respect to three-dimensional kinematic parameters, the maximum, minimum, and range-of-motion (ROM) values of the head, trunk, shoulder, elbow, forearm, and wrist exhibited no systematic or robust relationships with age. The few statistically significant associations identified were weak, sporadic, and inconsistent across joints and sides, precluding the identification of a clear age-dependent kinematic pattern.

Taken together, these findings suggest that the hand-to-mouth gesture exhibits a largely stable kinematic structure within this age range, with age-related effects primarily reflected in subtle refinements of movement dynamics (e.g., smoothness and velocity) rather than fundamental changes in motor organization.

## 4. Discussion

This study provides a sensor-based quantitative characterization of spatiotemporal and kinematic parameters of the hand-to-mouth movement performed in a standing position in a typically developing pediatric population aged 4 to 9 years using an adapted version of the “Rab Hand-to-Mouth protocol” for a standing position and an optoelectronic motion capture system. The proposed acquisition framework enabled standardized recording of physiological upper limb motion and the generation of consistent kinematic profiles under functionally relevant conditions. Furthermore, the influence of age and hand dominance on task execution was systematically evaluated. The main findings indicated that neither age nor manual dominance show limited and inconsistent effects on the kinematic organization of this functional task, indicating that the hand-to-mouth movement represents an early-acquired and highly automated motor pattern with pronounced bilateral symmetry at this age. These findings are consistent with previous studies on pediatric motor control development and provide preliminary descriptive data for a task commonly used in upper limb functional assessment [32,33].

### 4.1. Motion Capture-Based Interpretation of the Hand-to-Mouth Task

This study uses motion capture to characterize the hand-to-mouth movement performed while standing, emphasizing biomechanical modeling and signal-level interpretation. These findings expand on previous 3DMA of hand-to-mouth and hand-to-head gestures performed in a seated or quasi-static position. This task has previously been described as a highly stereotyped and repeatable motor behavior in pediatric and adult populations [7,12,14,33]. The results indicated that this gesture can be described as a low-variability, highly repeatable motor task with stable intersegmental coordination and consistent spatiotemporal organization in typically developing children aged 4 to 9 years.

The pronounced bilateral symmetry observed in joint kinematic waveforms and spatiotemporal parameters lends support to the use of a unified kinematic model for both limbs. This finding is consistent with earlier studies that reported minimal differences between dominant and non-dominant limbs during hand-to-mouth and reaching tasks in children and adults [11,33]. From a biomechanical modeling perspective, this symmetry allows for the aggregation of dominant and non-dominant limb data when constructing descriptive kinematic datasets that facilitate data handling and interpretation.

The few side-related differences detected at the head, trunk, and wrist levels were small and occurred only briefly. These variations are best interpreted as postural stabilization responses or residual variability associated with skin-mounted marker systems rather than meaningful differences in the motor organization of the task [32,34,35].

### 4.2. Relevance of Continuous Kinematic Analysis Using SPM

Integrating discrete kinematic metrics with continuous waveform analysis using SPM is a key methodological strength of this work. This approach is consistent with previous biomechanical studies that have highlighted the advantages of waveform-based analysis for detecting phase-specific differences in complex motor tasks [25,26]. Discrete variables summarize movement outcomes, whereas SPM preserves the temporal structure of joint angle signals, enabling phase-specific interpretation across the movement cycle.

The limited number of statistically significant SPM clusters between limbs indicates strong temporal coherence and waveform similarity. This finding is consistent with prior SPM-based investigations of upper limb kinematics, which reported highly reproducible joint angle trajectories in functional tasks. This consistency was maintained despite the increased degrees of freedom and postural demands associated with the standing position, supporting the feasibility and consistency of the adapted protocol from motion capture and signal quality standpoints. It is noteworthy that previous studies employing this protocol in a seated position did not incorporate SPM analyses, thereby highlighting the methodological novelty of the present study [36].

### 4.3. Spatiotemporal Metrics and Movement Efficiency

Spatiotemporal parameters such as movement time, the curvature index, the number of movement units, jerk, and velocity profiles provide insight into control efficiency and trajectory optimization. The low curvature index values and minimal number of movement units observed suggest smooth, goal-directed trajectories with limited online corrections, consistent with observations reported in previous studies on goal-directed reaching and feeding-related movements in pediatric populations [5,33,37].

Average jerk- and velocity-related parameters exhibited modest age-related trends without corresponding changes in joint kinematics. From a signal-processing perspective, this pattern reflects a refinement in movement smoothness and execution efficiency rather than biomechanical reorganization.

### 4.4. Influence of Age on the Stability of Kinematic Structures

Linear regression analyses of joint angles, range of motion, and inter-joint coordination indicate that the kinematic structure of hand-to-mouth movements remains largely unchanged from ages 4 to 9. This finding is consistent with previous developmental studies that reported early stabilization of kinematic patterns in function tasks [16]. This suggests that the task can be adequately characterized using a single joint reference kinematic template in typically developing children within this age range.

The low R^2^ values observed across regression models further support that age explains only a small proportion of variability, reinforcing the descriptive nature of these findings. Although some statistically significant associations were identified, these were not consistent across variables and should be interpreted cautiously considering their limited explanatory power.

Age-related changes were limited to execution-level parameters, such as smoothness and speed. This finding reinforces the idea that developmental effects primarily affect signal quality rather than the geometric organization of movement [38].

### 4.5. Limitations, Relevance for Clinical Motion Analysis Applications and Future Directions

One of the main limitations of this study is the small sample size, which was due to patients being limited solely to those with an age range of 4 to 9 years, as well as the low number of left-handed participants, which prevented stratified analyses by narrower age groups or laterality. The use of convenience sampling may also introduce selection bias. These factors limit the generalizability of the results and preclude the establishment of robust reference values across developmental stages. However, similar sample sizes have been reported in previous pediatric 3DMA studies that covered broader age ranges [11,12]. Second, the absence of a pathological comparison group prevents evaluation of the discriminative validity of the proposed kinematic framework. Therefore, the dataset should be interpreted as a preliminary descriptive reference. Third, although the acquisition protocol was standardized and based on widely used motion capture methodologies, no test–retest or inter-rater reliability analyses were conducted in this study. Consequently, the reliability and reproducibility of the measurements cannot be directly confirmed and should be formally evaluated in future work. Finally, the use of skin-mounted markers entails potential soft tissue artifacts, an inherent limitation of optical three-dimensional motion capture systems, and may affect kinematic accuracy [35].

Despite these limitations, the kinematic waveforms and spatiotemporal profiles reported here provide a preliminary descriptive dataset for 3DMA and biomechanical modeling. These profiles complement existing descriptive datasets and upper limb assessment frameworks reported in children [3,4,7]. Demonstrated symmetry and temporal consistency support the use of the hand-to-mouth task as a standardized acquisition approach for algorithm-based assessments, automated deviation detection, and longitudinal monitoring.

Future research should include larger cohorts to establish age-stratified normative databases and compare the reference values reported here with those of different clinical populations with neuromotor impairments. Comparisons between pathological movement patterns and the reference pattern described in this study may enhance the sensitivity of kinematic analysis for diagnostic purposes and for monitoring therapeutic interventions over time.

From a technological perspective, this dataset may serve as a preliminary reference for developing AI-driven applications. The consistency of the observed kinematic patterns could support machine learning models for movement classification, anomaly detection, and digital biomarker development. However, larger and more diverse datasets are needed to ensure robust model generalization.

## 5. Conclusions

This study provides a preliminary descriptive kinematic characterization of the hand-to-mouth movement in typically developing children aged 4 to 9 years using an adapted optoelectronic motion capture protocol. The results suggest that this gesture is a relatively stable and largely symmetrical motor pattern, with limited influence of age and hand dominance on its overall kinematic structure.

The proposed sensing framework enables the acquisition of physiological upper limb motion. These findings should be interpreted as preliminary descriptive data rather than definitive normative values.

Further research in larger and more diverse populations, including clinical cohorts, is required to confirm these observations and to determine their potential applicability in clinical and sensor-based assessment contexts.

## Figures and Tables

**Table 1:** Spatiotemporal parameter descriptions.

Parameter	Description
Movement time (s)	Time interval between the onset and completion of the gesture (outward and return phases).
Going Phase (%MC)	Phase corresponding to the forward movement of the hand from the predefined initial position toward the target (the mouth).
Adjusting Phase (%MC)	Phase corresponding to the fine motor adjustments required for accurate target localization (the mouth).
Return Phase (%MC)	Phase corresponding to the movement segment in which the hand returns from the target (the mouth) to the predefined initial position.
Index of Curvature	Movement straightness during the outward phase. A value of 1 indicates a perfectly straight movement.
Average Jerk (m/s^3^)	Movement smoothness. Lower values indicate a smoother, more continuous, and better-coordinated movement. The value decreases as smoothness increases.
Number of Movement Units	Number of online corrections performed during movement execution. A value of 2 indicates a confident movement with no corrections during either the outward or RP.
Mean Movement Velocity (m/s)	Mean velocity of the fingernail marker during the movement phase. An increase in this value indicates improved movement performance.
Peak Velocity (m/s)	Maximum movement velocity reached during the MC.
Skewness (%MC)	Time to peak velocity, expressed as a percentage of the MC. It reflects the temporal symmetry of the velocity profile.
Adjusting Sway (mm)	Adjustments performed to reach the target. This value decreases as movement accuracy increases.

Abbreviations: %MC = percentage of the cycle movement; m = meter; mm = millimeter; s = second.

**Table 2:** Comparison of kinematic parameters between the dominant and non-dominant side.

Kinematic Variables	Side	Max (Mean ± SD)	*p*-Value (Cohen’s d)	Min (Mean ± SD)	*p*-Value	ROM (Mean ± SD)	*p*-Value (Cohen’s d)
Head Lateral Bending	No Dom	1.88 ± 3.40	0.35 (−0.15)	−3.63 ± 4.45	0.40	5.51 ± 2.72	0.56 (−0.02)
	Dom	0.88 ± 3.32		−4.56 ± 3.44		5.44 ± 2.03	
Head Flex/Extension	No Dom	7.17 ± 11.29	0.35 (−0.15)	−1.72 ± 12.00 *	0.03 *	8.89 ± 4.19	0.06 (0.31)
	Dom	6.76 ± 10.92		−3.20 ± 11.71 *		9.96 ± 4.57	
Head Rotation	No Dom	3.87 ± 4.87	0.09 (−0.27)	−1.90 ± 5.79 *	0.03 *	5.77 ± 2.10	0.10 (−0.26)
	Dom	1.09 ± 5.32		−5.37 ± 4.67 *		6.47 ± 3.03	
Trunk Obliquity	No Dom	3.12 ± 2.58	0.26 (0.18)	−0.95 ± 1.69	0.79	4.07 ± 1.76 *	0.02 * (0.40)
	Dom	3.77 ± 2.30		−0.97 ± 1.90		4.75 ± 1.93 *	
Trunk Flex/Extension	No Dom	1.32 ± 10.21	0.27 (0.16)	−6.27 ± 9.71	0.71	7.58 ± 3.60	0.43 (0.16)
	Dom	1.81 ± 9.76		−6.21 ± 9.21		8.02 ± 3.95	
Trunk Rotation	No Dom	1.50 ± 5.63 *	0.01 * (0.41)	−4.20 ± 4.82 *	0.05 *	5.70 ± 2.22	0.15 (0.23)
	Dom	5.43 ± 4.55 *		−0.89 ± 5.22 *		6.32 ± 2.98	
Shoulder Ab/Adduction	No Dom	11.35 ± 4.39	0.14 (0.24)	−2.82 ± 5.36	0.16	14.17 ± 4.70	0.59 (0.09)
	Dom	12.87 ± 5.58		−1.66 ± 6.14		14.53 ± 5.01	
Shoulder Flex/Extension	No Dom	34.43 ± 14.75	0.73 (0.06)	−12.99 ± 9.87	0.12	47.43 ± 12.61	0.13 (0.25)
	Dom	34.85 ± 13.48		−14.71 ± 9.66		49.56 ± 11.14	
Shoulder Rotation	No Dom	30.17 ± 7.05	0.61 (−0.08)	−1.07 ± 13.65	0.71	31.24 ± 13.52	0.97 (0.01)
	Dom	29.54 ± 7.08		−1.77 ± 12.34		31.31 ± 13.02	
Elbow Flex/Extension	No Dom	136.47 ± 3.67	0.83 (0.03)	24.34 ± 6.54	0.16	112.12 ± 6.77	0.28 (−0.18)
	Dom	136.60 ± 4.25		25.75 ± 5.58		110.85 ± 6.47	
Forearm Prono/Supination	No Dom	25.44 ± 10.48	0.34 (−0.16)	−37.30 ± 11.94	0.33	62.74 ± 12.39	0.95 (0.01)
	Dom	23.59 ± 11.45		−39.27 ± 11.07		62.86 ± 13.29	
Wrist Radial–Ulnar Deviation	No Dom	−0.25 ± 7.01	0.16 (−0.23)	−13.81 ± 6.36	0.83	13.56 ± 4.29 *	0.03 * (−0.28)
	Dom	−1.75 ± 7.49		−13.88 ± 5.70		12.13 ± 5.07 *	
Wrist Dorsi/Palmar Flexion	No Dom	12.78 ± 7.88	0.97 (−0.01)	−2.07 ± 8.16	0.63	14.85 ± 4.52	0.47 (−0.12)
	Dom	12.73 ± 7.79		−1.46 ± 8.80		14.18 ± 3.93	

* *p* < 0.05. Max = maximum; Min = minimum; ROM = range of movement; Dom = dominant; No Dom = non-dominant.

**Table 3:** Linear regression results for the association between age and kinematic and spatiotemporal parameters.

Kinematic Variables		Side	Coef	CI_lower	CI_upper	*p*_valor	R^2^
Head Lateral Bending	Max	Dom	0.29	−0.40	0.98	0.40	0.02
		No Dom	−0.20	−0.91	0.51	0.57	0.01
	Min	Dom	0.64	−0.05	1.33	0.07	0.09
		No Dom	0.33	−0.60	1.25	0.48	0.01
	ROM	Dom	−0.34	−0.75	0.07	0.10	0.07
		No Dom	−0.53	−1.07	0.01	0.05 *	0.10
Head Flex/Extension	Max	Dom	>−0.05	−2.34	2.24	0.96	0.00
		No Dom	0.08	−2.28	2.45	0.94	0.00
	Min	Dom	0.59	−1.86	3.03	0.63	0.01
		No Dom	1.09	−1.40	3.58	0.38	0.02
	ROM	Dom	−0.64	−1.57	0.30	0.18	0.05
		No Dom	−1.01	−1.82	−0.19	0.02 *	0.15
Head Rotation	Max	Dom	−0.97	−2.04	0.10	0.07	0.08
		No Dom	0.65	−0.35	1.65	0.19	0.05
	Min	Dom	−0.38	−1.35	0.59	0.43	0.02
		No Dom	0.96	−0.21	2.14	0.10	0.07
	ROM	Dom	−0.59	−1.20	0.01	0.05 *	0.10
		No Dom	−0.31	−0.74	0.12	0.15	0.06
Trunk Obliquity	Max	Dom	0.12	−1.93	2.16	0.91	0.00
		No Dom	−0.22	−2.36	1.92	0.84	0.00
	Min	Dom	0.79	−1.12	2.71	0.41	0.02
		No Dom	0.59	−1.44	2.62	0.56	0.01
	ROM	Dom	−0.68	−1.47	0.12	0.09	0.07
		No Dom	−0.81	−1.52	−0.11	0.03 *	0.13
Wrist Radial–Ulnar Deviation	Max	Dom	0.74	−0.81	2.29	0.34	0.02
		No Dom	1.58	0.20	2.95	0.03 *	0.13
	Min	Dom	0.84	−0.33	2.00	0.15	0.05
		No Dom	0.82	−0.49	2.12	0.21	0.04
	ROM	Dom	−0.09	−1.16	0.97	0.86	0.00
		No Dom	0.76	−0.10	1.63	0.08	0.08

* Statistically significant results (*p* < 0.05) are indicated; however, low R2 values reflect limited explanatory power of age. Max = maximum; Min = minimum; ROM = range of movement; Dom = dominant; No Dom = non-dominant.

## Data Availability

The data that support the findings of this study are not publicly available because they contain confidential patient information. Access to the data may be considered upon reasonable requests to the corresponding author and with approval from the institutional ethics committee.

## Abbreviations

The following abbreviations are used in this manuscript:

3DMA	Three-Dimensional Movement Analysis
ADLs	Activities of Daily living
TD	Typical Developmental
OLS	Ordinary Least Squares
ROM	Range of Movement
SPM	Statistical Parametric Mapping
EHI	Edinburgh Handedness Inventory
%MC	Percentage of the Movement Cycle
m	Meter
mm	Millimeter
s	Second
Max	Maximum
Min	Minimum
Dom	Dominant
No Dom	Non-Dominant
